# The “EndoBubbloMeter”: a novel orientation method to facilitate straight tunneling in peroral endoscopic myotomy

**DOI:** 10.1055/a-2686-7964

**Published:** 2025-09-11

**Authors:** Satoshi Abiko, Haruhiro Inoue, Kei Ushikubo, Kazuki Yamamoto, Yohei Nishikawa, Ippei Tanaka, Naoya Sakamoto

**Affiliations:** 1 Digestive Disease Center, Showa Medical University Koto Toyosu Hospital, Tokyo, Japan; 2 Department of Gastroenterology and Hepatology, Hokkaido University Hospital, Sapporo, Japan

In peroral endoscopic myotomy (POEM), precise endoscope positioning at the 2 o’clock position of the lower esophageal sphincter (LES) – between the anterior and posterior sling fibers while avoiding injury – is critical to minimize postoperative gastroesophageal reflux 1 2 3 and ensure long-term efficacy. However, maintaining a straight submucosal tunnel and aligning the scope to this axis can be challenging, especially for novice operators. Overrotation may cause deviation into the sling fibers. To address this, we developed the EndoBubbloMeter, a novel orientation method inspired by the principle of a spirit level. By visualizing air bubbles within the fluid-filled transparent cap at the tip of the endoscope, this technique helps maintain horizontal alignment during tunnel dissection ( Fig. 1 **a–c** ).

The video presents a case of peroral endoscopic myotomy performed using the EndoBubbloMeter.Video 1

The EndoBubbloMeter was employed in a 73-year-old woman undergoing POEM. A submucosal entry was made at the 2 o’clock position of the esophagus, and dissection proceeded in that direction. Air bubbles were kept centered to ensure horizontal orientation, allowing for a straight tunnel. Upon reaching the 2 o’clock position of the LES, the endoscope entered the sweet spot with a snug sensation. Dissection was then continued along the lesser curvature of the stomach while maintaining proper orientation. Using the double-scope technique 4 with a retroflexed view, the tunnel’s length and its position relative to the sling fibers were confirmed ( Fig. 2 **a** ). The same findings were also confirmed using a forward view ( Fig. 2 **b** ). The tunnel was confirmed to pass through the 2 o’clock position of the LES, with the endoscopic view rotated to place the LES’s 2 o’clock direction at 12 o’clock on the screen. A straight tunnel was successfully created from the esophageal side, reaching the 2 o’clock position of the LES, and then continued without injuring the sling fibers, exiting into the lesser curvature of the stomach ( Video 1 ).

**Fig. 1:**
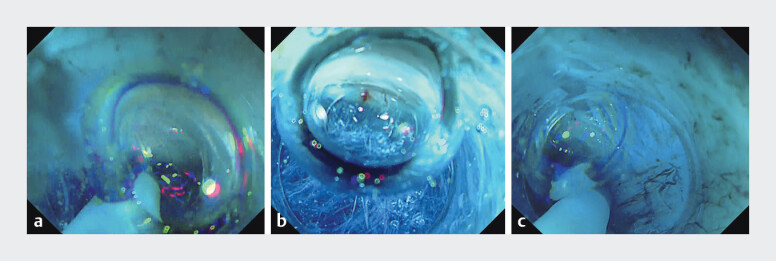
Endoscopic images showing the position of the air bubbles within the transparent distal cap using the EndoBubbloMeter. **a** The bubbles are shifted to the right side, indicating that the endoscope is tilted to the left. **b** The bubbles are centered, indicating that a horizontal orientation is maintained. This is the ideal view for performing submucosal dissection at the 2 o’clock position. **c** The bubbles are shifted to the left side, indicating that the endoscope is tilted to the right.

**Fig. 2:**
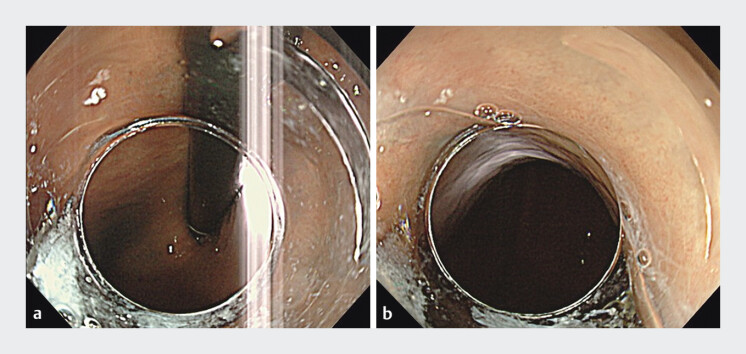
Endoscopic images showing the double-scope technique with a retroflexed and forward views. **a** Using the double-scope technique with a retroflexed view, the tunnel’s length and its position relative to the sling fibers were confirmed. **b** The same findings were also confirmed using a forward view.

Endoscopy_UCTN_Code_TTT_1AO_2AP

## Notes

Endoscopy E-Videos https://eref.thieme.de/e-videos
*E-Videos* is an open access online section of the journal *Endoscopy* , reporting on interesting cases and new techniques in gastroenterological endoscopy. All papers include a high-quality video and are published with a Creative Commons CC-BY license. Endoscopy E-Videos qualify for HINARI discounts and waivers and eligibility is automatically checked during the submission process. We grant 100% waivers to articles whose corresponding authors are based in Group A countries and 50% waivers to those who are based in Group B countries as classified by Research4Life (see: https://www.research4life.org/access/eligibility/ ). This section has its own submission website at https://mc.manuscriptcentral.com/e-videos .
